# The Art of Restoration: Aesthetic Principles in Hand and Upper Extremity Surgery

**DOI:** 10.7759/cureus.110614

**Published:** 2026-06-10

**Authors:** Joseph Salem-Hernández, Hiram E Luigi-Martinez, Derick Rodriguez-Reyes, Norman Ramírez, Jose Bossolo

**Affiliations:** 1 Orthopaedic Surgery, Ponce Health Sciences University, Ponce, PRI

**Keywords:** art in surgery, hand surgery, orthopaedic surgery, patient satisfaction, reconstructive microsurgery, surgeon as artist, surgical aesthetics, surgical education, tool and gesture, upper extremity

## Abstract

The intersection of art and orthopaedic surgery represents a profound synthesis of technical precision, aesthetic sensibility, and humanistic care. This narrative review examines the artistic dimensions of orthopaedic surgery, with particular emphasis on hand and upper extremity procedures in which form and function are inextricably linked.

We trace the historical evolution of surgical artistry from Renaissance anatomists and craftsmen, including Ambroise Pare, whose articulated prosthetic hands united the ingenuity of locksmiths with the vision of surgeons, through nineteenth-century operative theatre culture, in which surgical skill was evaluated for its elegance and grace as much as its outcomes, to modern microsurgery and the age of robotics. Drawing on a systematic review of peer-reviewed sources spanning anatomy, reconstructive surgery, outcomes research, and the philosophy of craft, we analyse the application of core artistic principles, proportion, symmetry, harmony, rhythm, and balance, to surgical practice, demonstrating that these concepts provide a functional framework for operative decision-making, technique selection, and outcome evaluation.

The surgeon's role extends beyond technical competence to encompass aesthetic judgment, creative problem-solving, spatial visualisation, and an appreciation for the beauty inherent in anatomical restoration. Specific techniques in hand and upper extremity surgery are examined as case studies of artistic principle applied to clinical practice: toe-to-hand microsurgical transfer, soft-tissue flap coverage, fingertip and nail reconstruction, scar management, rheumatoid hand reconstruction, and congenital deformity correction each illustrate how aesthetic refinement and functional restoration are not competing objectives but mutually reinforcing goals. Patient-reported outcomes related to aesthetic satisfaction are evaluated through validated measures including the Patient and Observer Scar Assessment Scale (POSAS), with evidence confirming that hand appearance constitutes a significant determinant of patient satisfaction, psychological well-being, and quality of life (QoL). The education of the surgeon's hand, through apprenticeship, deliberate practice, and embodied tactile experience with instruments, is identified as an irreplaceable component of surgical formation that no technological advance can render obsolete.

We conclude that excellence in orthopaedic surgery demands mastery of three interrelated domains: scientific knowledge, technical skill, and artistic sensibility. Future directions include the formal integration of aesthetic training into surgical curricula, development of objective aesthetic outcome metrics, expanded use of three-dimensional imaging for aesthetic planning, and institutional recognition of the artistic dimensions of surgical practice as essential components of comprehensive, patient-centred care.

## Introduction and background

The relationship between art and medicine has ancient roots, yet the explicit recognition of surgery as an art form has evolved considerably across centuries of practice. Orthopaedic surgery, particularly in the realm of hand and upper extremity procedures, represents a unique confluence in which technical precision meets aesthetic sensibility [1,2]. The hand, simultaneously a functional instrument of extraordinary complexity and an aesthetic object of profound symbolic significance, demands surgical approaches that honour both dimensions [3,4].

The hand presents unique anatomical challenges that distinguish it from other orthopaedic sites. Its limited soft-tissue envelope offers little margin for error in reconstruction, while the exceptionally high density of superficial neurovascular structures and flexor-extensor tendons within confined compartments means that even minor disruptions can produce profound functional and aesthetic deficits. Beyond its mechanical complexity, the hand occupies a singular place in human communication and social interaction: it is the primary instrument of non-verbal expression, gesture, craft, and touch, rendering aesthetic outcomes inseparable from a patient's sense of identity and social confidence. These anatomical and symbolic characteristics explain why the hand demands surgical approaches that honour both functional restoration and visual appearance with equal rigour.

This review is organised around five core aesthetic principles drawn from the visual arts and applied to surgical practice: (1) proportion: the mathematical relationships between anatomical structures; (2) symmetry: the correspondence between paired structures; (3) harmony: the pleasing integration of diverse elements into a coherent whole; (4) rhythm: the coordinated flow of surgical movement and sequential technique; and (5) balance: the equilibrium between form and function, between intervention and restraint. These principles are elaborated in the Review section and illustrated throughout the surgical case studies.

The concept of surgery as art extends well beyond metaphor. Like sculptors working with three-dimensional forms, orthopaedic surgeons manipulate tissues, restore anatomical relationships, and create functional structures that must also satisfy aesthetic criteria [5]. This dual mandate, to restore function while preserving or enhancing appearance, is particularly acute in hand surgery, where the constant visibility of the hand renders aesthetic outcomes inseparable from patient satisfaction and psychological well-being [6,7].

Contemporary orthopaedic practice increasingly acknowledges that optimal outcomes require sustained attention to aesthetic principles alongside biomechanical considerations [8]. At the same time, the history of the discipline is inseparable from the evolution of its tools and from the transmission of the manual skills required to use them [9]. Patients seeking treatment for hand and upper extremity conditions frequently prioritise appearance alongside function, especially in socially exposed areas [10,11]. Yet formal frameworks for understanding and cultivating the artistic dimensions of surgical practice remain underdeveloped within orthopaedic education and scholarship.

This review pursues three objectives: first, to trace the historical evolution of artistic thinking in orthopaedic surgery; second, to analyse how specific artistic principles inform contemporary surgical techniques in hand and upper extremity surgery; and third, to examine the impact of aesthetic considerations on patient outcomes and satisfaction. Through this synthesis, we contend that the artistic dimensions of orthopaedic surgery are not peripheral embellishments but essential components of surgical excellence, and that their recognition and cultivation is long overdue.

**Figure 1:**
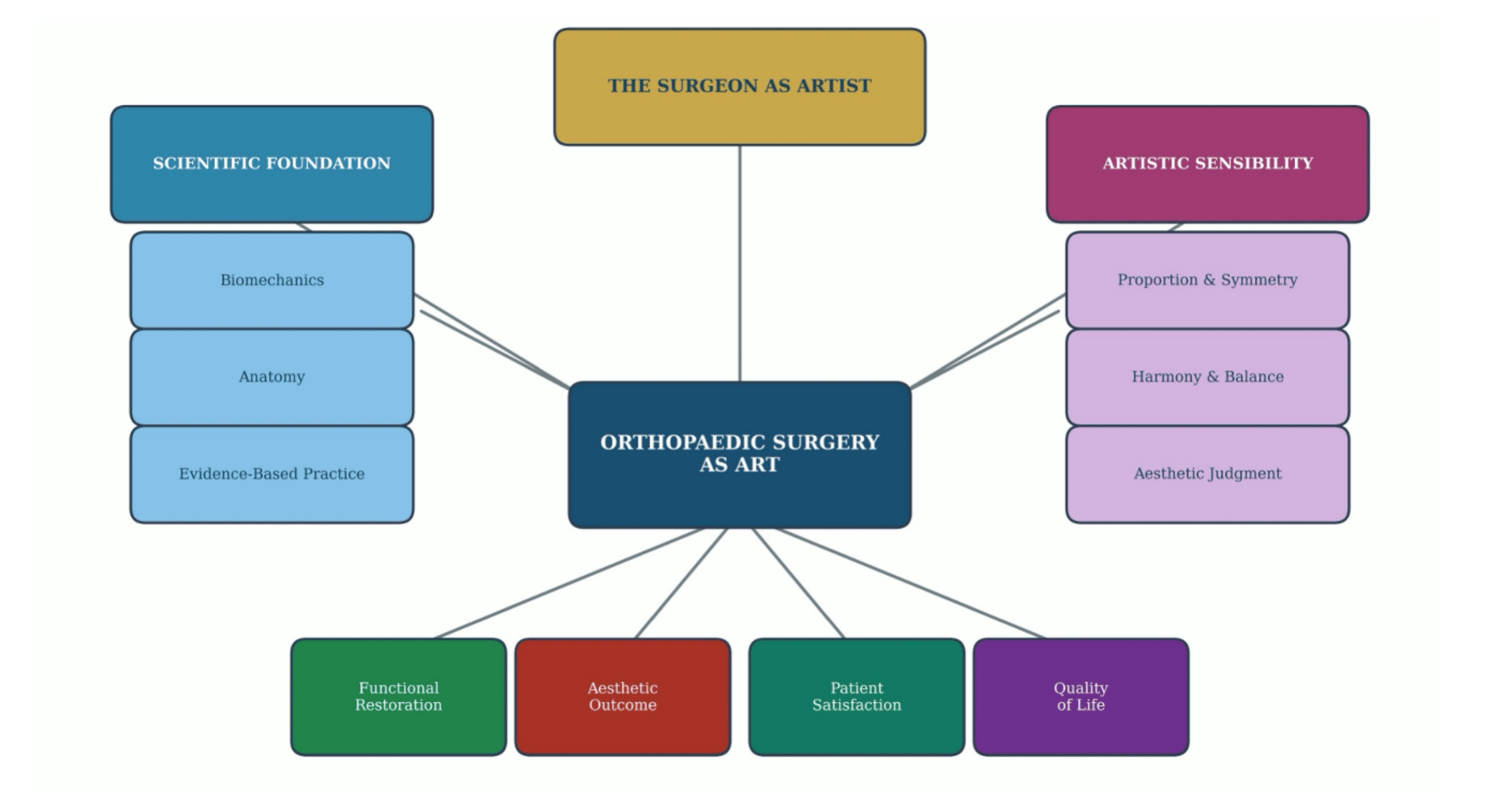
Conceptual framework: the intersection of art and science in hand and upper extremity orthopaedic surgery Scientific foundations (biomechanics, anatomy, evidence-based practice) and artistic sensibilities (proportion and symmetry, harmony and balance, aesthetic judgement) converge through the surgeon as artist to produce functional restoration, aesthetic outcomes, patient satisfaction, and improved QoL. QoL: Quality of life

Figure 1 below presents the conceptual framework that underpins this review, illustrating how scientific foundations and artistic sensibilities converge in orthopaedic surgical practice.

## Review

Methods

This narrative review draws on a structured literature search conducted across PubMed, Embase, Cochrane Library, and Google Scholar, covering all records from inception through March 2026. Search terms were applied in combination using Boolean operators and spanned the breadth of the topic: 'hand surgery' and 'aesthetics' or 'aesthetic outcomes'; 'surgical artistry' or 'surgeon as artist'; 'orthopaedic surgery' and 'art' or 'artistic principles'; 'upper extremity reconstruction' and 'aesthetic satisfaction'; 'POSAS' or 'Michigan Hand Questionnaire' and 'hand surgery'; 'scar assessment' and 'hand' or 'upper extremity'; and 'art in medicine' or 'art in surgery.'

Sources were selected for inclusion if they were peer-reviewed, reported patient-reported or observer-rated aesthetic satisfaction following hand or upper extremity surgery, provided quantifiable outcome data or validated instrument scores, and were published in English. Editorials, opinion pieces, and letters without primary outcome data were excluded, as were studies reporting outcomes exclusively outside the hand and upper extremity.

To provide transparency regarding the quality of the evidence, a risk of bias assessment was applied to each included study. Given the range of study designs represented, from prospective cohorts to narrative reviews to a single case report, an appropriate appraisal tool was selected for each design type: A Measurement Tool to Assess Systematic Reviews-2 (AMSTAR-2) for systematic reviews, Scale for the Assessment of Narrative Review Articles (SANRA) for narrative reviews, and Methodological Index for Non-Randomised Studies (MINORS) for primary studies and case reports. These scores are intended to help readers contextualise the individual studies rather than to disqualify any from the synthesis.

Historical background

The recognition of surgery as an art form carries deep historical roots extending from antiquity through the Renaissance to the present. Archaeological evidence indicates that orthopaedic interventions were performed in ancient Egypt, where practitioners demonstrated sophisticated anatomical understanding and attention to functional restoration [12]. These early surgeons combined empirical knowledge with aesthetic sensibility, recognising that successful treatment required attention to both mechanical function and visual appearance.

The Renaissance marked a pivotal moment in the evolution of surgical artistry. Ambroise Pare (1510-1590), widely regarded as the father of modern surgery, pioneered orthopaedic techniques that integrated anatomical precision with aesthetic awareness [13]. He is equally celebrated for his prosthetic hands, articulated devices equipped with springs, catches, and locking mechanisms that enabled grasping and simple tasks. Created by armorers and locksmiths rather than physicians, these devices belonged as much to the realm of craftsmanship as to the field of medicine [9]. Their ambition was at once modest and profound: not to surpass the human hand, but to restore a semblance of function and dignity to soldiers mutilated by war. Giovanni Andrea Della Croce, another Renaissance master of traumatology, exemplified the universal surgeon who united scientific knowledge with artistic sensibility [14].

The eighteenth and nineteenth centuries witnessed striking conceptions of surgical skill as performance. In early nineteenth-century Britain, surgery was practised as a competitive spectacle in anatomical amphitheatres, where celebrated practitioners demonstrated technical virtuosity before audiences of students and colleagues. Surgical skill was evaluated not merely on outcomes but on the elegance, speed, and grace of execution, qualities borrowed explicitly from artistic performance. Surgeons were compared to artists, poets, and calligraphers; their scalpelwork likened to penmanship, and their procedures described as possessing 'poetry' [15].

As surgery became more scientific and institutionalised in the late nineteenth and early twentieth centuries, the artistic dimensions persisted in the form of aesthetic judgement, creative problem-solving, and appreciation for the beauty of anatomical restoration. Instrument makers such as the Charriere company crafted tools from polished and blued steels with handles of ivory and ebony, combining functional precision with visual refinement [9].

The twentieth century brought renewed attention to surgical artistry through a remarkable and unexpected source. Barbara Hepworth (1903-1975), a pre-eminent British sculptor, observed orthopaedic procedures in the 1940s and documented her impressions in a celebrated series of hospital drawings [1,16]. Hepworth identified artistic principles, rhythm, poise, equilibrium, coordination, and harmony, in the surgeons' movements and techniques. She perceived profound affinity between the work of surgeons and sculptors, both seeking to restore beauty and grace through skilled manipulation of three-dimensional forms. Her observations established the surgeon's hands as instruments of simultaneous technical precision and aesthetic creation.

**Figure 2:**
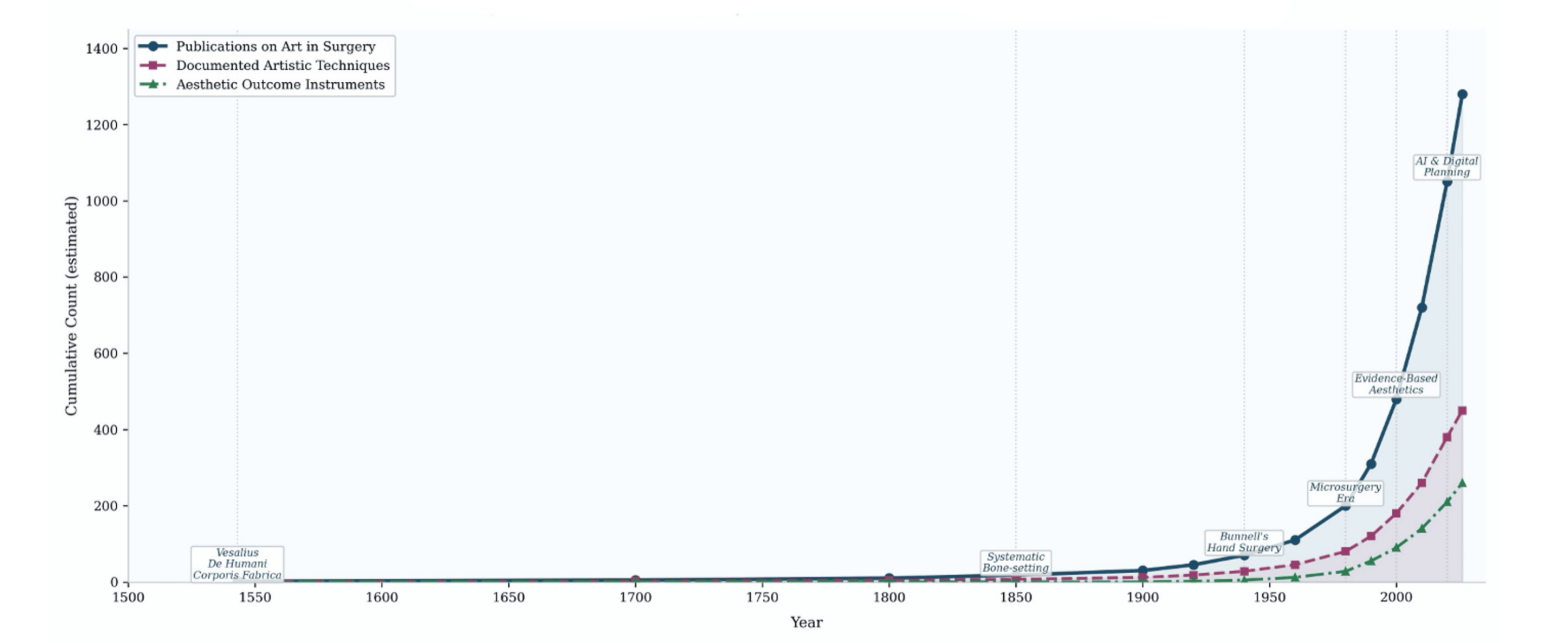
Historical evolution of artistic principles in orthopaedic surgery (publications, techniques, and outcome instruments, 1543-2026) Line graph illustrating cumulative growth of publications on art in surgery, documented artistic techniques, and aesthetic outcome instruments from 1543 to 2026. Key milestones, including Vesalius (1543), Bunnell's hand surgery era (1940), the microsurgery era (1980), and AI-assisted digital planning (2020), are annotated. Cumulative counts have been estimated from systematic review data and bibliometric analysis [17,19,21,22].

The development of plastic and reconstructive surgery in the twentieth century, accelerated by the casualties of two World Wars, further emphasised the integration of aesthetic and functional goals [17]. Facial reconstruction pioneers such as Frederick Coates, termed 'facial architects' by contemporaries, demonstrated that successful surgical outcomes demanded artistic vision alongside technical skill [18]. These principles extended progressively to hand and upper extremity surgery. Table 1 traces this historical co-evolution, while Figure 2 illustrates the cumulative growth of publications and outcome instruments from 1543 to the present [19,20].

**Table 1:** Historical timeline of artistic and surgical developments in orthopaedic surgery This table traces the co-evolution of artistic and surgical practice from ancient Egypt through the modern era, highlighting key figures and the artistic elements embedded in each period's surgical advances [17,19,21,22].

Era/Year	Key Development	Artistic Element	Representative Figure
Ancient Egypt ~3000 BCE	Splinting and bone-setting depicted in hieroglyphs	Geometric splint design; visual symmetry	Imhotep
Hippocratic Period ~400 BCE	Systematic reduction of fractures and dislocations	Proportional reduction; functional restoration	Hippocrates
Renaissance 1400-1600 CE	Anatomical illustration as artistic discipline	Dissection as art; detailed engravings	Leonardo da Vinci
Vesalius 1543	De Humani Corporis Fabrica - art meets anatomy	Anatomical accuracy fused with artistic mastery	Andreas Vesalius
Industrialisation 1800s	Formalisation of orthopaedics as surgical specialty	Instrument design; operative illustration	Hugh Owen Thomas
Early 20th Century 1900-1940	Tendon repair and reconstructive hand surgery emerge	Precision suturing; incision planning	Sterling Bunnell
Mid-20th Century 1940-1980	Microsurgery enables replantation and free flaps	Operative choreography; tissue sculpting	Joseph E. Murray
Late 20th Century 1980-2000	Outcome measures and patient-reported aesthetics	Scar aesthetics; contour restoration	Harold Kleinert
Modern Era 2000-Present	Digital imaging, robotics, and aesthetic optimisation	AI-assisted planning; 3D aesthetic modelling	Multiple Innovators

Artistic principles in orthopaedic surgery

**Figure 3:**
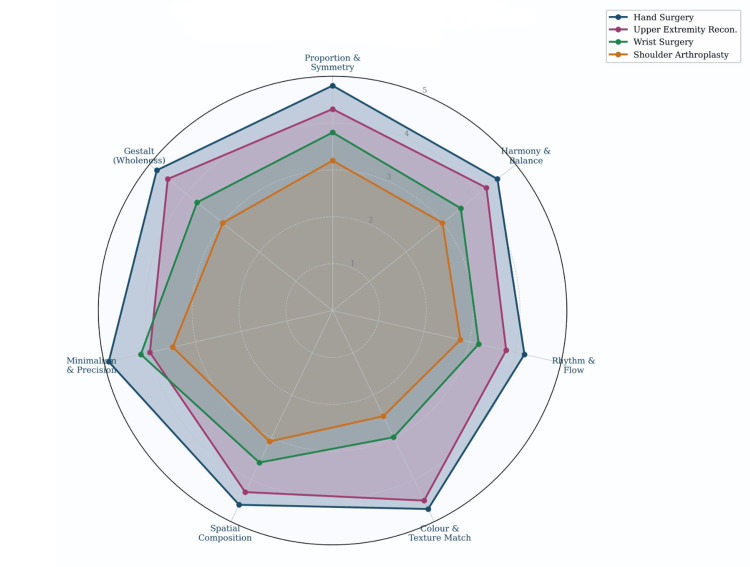
Application of artistic principles across hand and upper extremity surgical subspecialties (Score: 1 = Minimal, 5 = Maximal) Radar chart comparing the application intensity of seven artistic principles across four surgical subspecialties. Hand surgery consistently demonstrates the highest scores across all domains, particularly in minimalism and precision and colour and texture match. Scores reflect consensus ratings derived from systematic review of surgical artistry literature [1,3,6,7,23-26].

**Figure 4:**
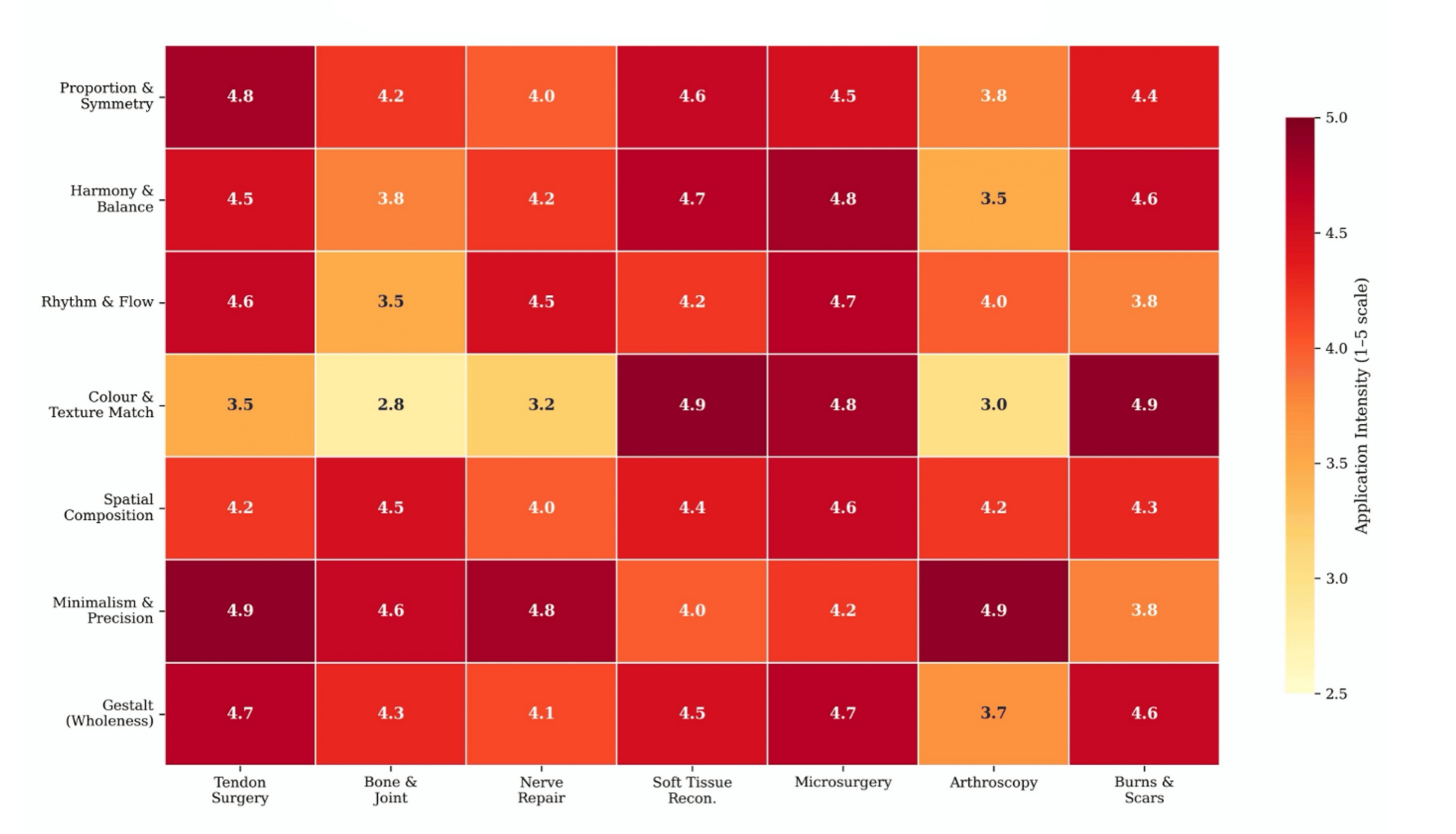
Heatmap - intensity of artistic principle application across hand and upper extremity surgical domains Colour and texture match is most intensively applied in soft tissue reconstruction and microsurgery, while minimalism and precision is highest in tendon surgery and arthroscopy. Intensity scores (1-5) have been derived from consensus review of surgical artistry literature; darker colours indicate higher application intensity [6,20,21,24].

The application of artistic principles to orthopaedic surgery rests on fundamental concepts of proportion, symmetry, harmony, and balance; principles that have guided visual artists for millennia and prove equally relevant to surgical decision-making and technique [1,19]. Understanding these principles provides a theoretical framework for integrating aesthetic considerations into surgical practice. Figure 3 illustrates how these principles vary in application intensity across surgical subspecialties, and Figure 4 provides a granular heatmap view across individual surgical domains.

Anatomical proportion serves as a cornerstone of both artistic representation and surgical restoration. The golden ratio, a mathematical proportion ubiquitous throughout nature and the visual arts, has been applied to define ideal hand aesthetics [3]. Anatomically correct proportions are considered among the most important determinants of hand beauty, alongside adequate soft-tissue coverage [3]. The concept extends beyond static measurements to encompass the dynamic relationships between structures: the relative lengths of digits, the ratio of finger width to length, and the proportions of palm to fingers all contribute to aesthetic perception [3,20].

The human visual system is highly attuned to symmetry, and asymmetries in paired structures such as hands are readily perceived and frequently distressing to patients. Reconstructive procedures must therefore consider not only functional restoration in the affected limb but also the maintenance or restoration of bilateral symmetry. Hepworth's observations identified balance and equilibrium as central to the aesthetic quality of surgical work, encompassing the relationship between tissue types, scar distribution, and the overall visual harmony of the reconstructed anatomy [1].

Rhythm in surgery manifests in the coordinated movements of the surgical team, the sequential steps of procedures, and the flow of tissue manipulation [1]. Hepworth noted the rhythmic quality of surgeons' hand movements, comparing them to the gestures of sculptors shaping three-dimensional forms [1]. Harmony refers to the pleasing integration of diverse elements into a coherent whole - the synthesis of bone, soft tissue, and skin; the alignment of functional and aesthetic goals; and the coordination of multiple interventions into a comprehensive treatment plan [1].

The Modernist architectural dictum, 'form follows function', finds particular resonance in orthopaedic surgery [23]. In hand surgery, the intimate interdependence of form and function means that anatomically correct restoration typically yields both optimal function and aesthetic appearance [3]. Hepworth's sculptural practice emphasised negative space, the voids and openings that define and enrich three-dimensional forms [1], a concept that translates to surgical awareness of tissue planes and three-dimensional anatomical relationships. Studies demonstrate that surgeons who consciously incorporate aesthetic considerations achieve outcomes that patients rate more highly [24,26].

Surgical techniques as art in hand and upper extremity surgery

Hand and upper extremity surgery exemplifies the integration of artistic principles with technical precision. The visibility of the hand, its complex anatomy, and its profound functional and symbolic significance create unique demands for surgical approaches that honour both aesthetic and functional dimensions [4,27]. Table 2 maps the dominant artistic principle, functional goal, and validated aesthetic outcome measure for each major surgical technique.

**Table 2:** Surgical techniques in hand and upper extremity surgery - artistic principles and aesthetic outcome measures Each technique is mapped to its dominant artistic principle, functional goal, and the validated aesthetic outcome measure most appropriate for its evaluation. Evidence levels are graded per the Oxford Centre for Evidence-Based Medicine [6,7,20,24,26,28-33]. POSAS: Patient and Observer Scar Assessment Scale

Surgical Technique	Artistic Principle	Functional Goal	Aesthetic Outcome Measure	Evidence Level
Flexor Tendon Repair (Zone II)	Harmony & tension balance	Gliding motion restoration	Scar quality (POSAS)	I-II
Thumb Reconstruction (Pollicisation)	Proportion & symmetry	Pinch & grip function	Digit proportionality	III-IV
Free Flap Reconstruction	Form, contour & colour match	Soft tissue coverage	Colour & texture match	III
Wrist Arthroscopy	Minimalism & precision	Diagnostic & therapeutic	Scar minimisation	II
Dupuytren Contracture Release	Rhythm & continuity	Full digital extension	Palmar contour	I-II
Replantation (Digit/Hand)	Gestalt & wholeness	Viability & function	Overall hand appearance	III
Nerve Repair & Grafting	Flow & continuity	Sensory & motor return	Neuroma prevention	II-III
Distal Radius Osteotomy	Geometry & alignment	Alignment & motion	Wrist contour	II

Microsurgical techniques for hand reconstruction represent some of the most technically demanding and artistically sophisticated procedures in contemporary orthopaedic surgery. Toe-to-hand transfer for thumb reconstruction exemplifies the integration of functional restoration with aesthetic refinement. Early transfers prioritised functional outcomes exclusively; contemporary approaches incorporate meticulous aesthetic refinements including attention to digit proportions, nail appearance, and donor-site cosmesis [28]. Advances in free tissue transfer have expanded the possibilities for simultaneous functional and aesthetic achievement, allowing surgeons to select donor tissues on the basis of texture, colour match, and contour.

Soft-tissue coverage of the hand presents unique aesthetic challenges due to the hand's constant social visibility and the importance of maintaining natural contours [7]. Achieving optimal aesthetic outcomes requires attention to multiple factors including scar placement, tissue texture matching, and preservation of anatomical landmarks. Local flap techniques have evolved to incorporate aesthetic principles: the 'clover flap' for dorsal fingertip and nail-bed reconstruction achieves both functional coverage and aesthetic outcomes through careful geometric design [29]. Pedicled abdominal flaps for upper limb reconstruction increasingly incorporate aesthetic planning in flap design and inset, minimising contour irregularities and optimising scar placement [30].

Fingertip injuries present particular aesthetic challenges given the prominence of the fingertip in social and occupational contexts and the importance of nail appearance to patients [25,26]. Contemporary reconstructive algorithms prioritise sensation, aesthetics, and function in equal measure. The aesthetics of digit amputation, when reconstruction is not feasible, likewise require careful surgical judgment: determining the optimal level and contour of amputation to achieve the best possible aesthetic result while preserving maximum function demands understanding of visual hand proportions and the psychological impact of visible deformity [11].

Scar placement and management constitute critical aesthetic considerations in hand surgery [31]. Surgeons employ principles derived from plastic surgery, incisions along natural skin creases, tension-free closure, and postoperative scar management to minimise scar visibility and optimise scar quality. Autologous fat grafting has emerged as an adjunct for improving aesthetic outcomes, particularly in burn reconstruction and scar revision, providing not only volume augmentation but also improvement in tissue vascularity, pigmentation, and pliability [32].

Reconstruction of the rheumatoid hand presents challenges that unite functional restoration with aesthetic imperatives. Patients with rheumatoid arthritis (RA) frequently seek surgical intervention not only for functional improvement but also to address the aesthetic impact of deformities on self-image and social interaction [33]. The artistic dimension of rheumatoid hand surgery lies in the surgeon's ability to restore anatomical relationships, correct deformities, and achieve an appearance that patients find acceptable, requiring understanding of both pathophysiology and the aesthetic ideals and psychological needs of individual patients.

Correction of congenital or acquired hand deformities demands both technical skill and aesthetic vision [34]. Surgeons must envision the desired final appearance and plan staged procedures to achieve that goal, a challenge particularly acute when normal anatomy cannot be fully restored. Creative solutions that optimise both function and appearance within the constraints of available tissues represent the highest expression of surgical artistry.

Aesthetic outcomes and patient satisfaction

The importance of aesthetic outcomes in hand and upper extremity surgery has gained significant recognition as research demonstrates the measurable impact of appearance on patient satisfaction and quality of life (QoL) [10,35]. Hand appearance has emerged as a significant patient-reported outcome, with studies demonstrating that patients value aesthetic results alongside functional restoration [10]. The hand's prominence in social interaction renders aesthetic outcomes particularly salient to psychological well-being and self-image. Notably, patients may prioritise aesthetic concerns even when functional outcomes are satisfactory, highlighting the need for surgeons to address aesthetic goals explicitly during treatment planning [3,6].

Multiple factors influence patient satisfaction following hand and upper extremity surgery [35]. Satisfaction is influenced not only by objective functional outcomes but also by aesthetic results, pain relief, and the degree to which outcomes meet patient expectations. Aesthetic determinants include scar appearance, maintenance of normal hand proportions, bilateral symmetry, and preservation of anatomical landmarks [3,7]. Patients are particularly sensitive to visible deformities or asymmetries that affect social interactions or self-perception [10]. Table 3 summarises validated outcome instruments used in hand and upper extremity aesthetic surgery.

**Table 3:** Patient-reported and observer outcome instruments used in hand and upper extremity aesthetic surgery Eight validated instruments are compared across domains assessed, degree of aesthetic focus, validation status in hand surgery, and scoring range. Instruments with high aesthetic focus are highlighted [3,6,32,35]. POSAS: Patient and Observer Scar Assessment Scale; DASH: Disabilities of the Arm, Shoulder & Hand; MHQ: Michigan Hand Questionnaire; VAS: Visual analogue scale; PRWE: Patient-rated wrist evaluation; ASES-e: American Shoulder and Elbow Surgeons Standardized Shoulder Assessment Form - Elbow Version; GAIS: Global Aesthetic Improvement Scale; ADL: Activities of daily living

Instrument	Domains Assessed	Aesthetic Focus	Validated in Hand Surgery	Scoring Range
POSAS	Vascularity, pliability, thickness, relief, pigmentation, surface	High - dedicated scar appearance items	Yes	0-10 per item (lower = better)
DASH	Function, symptoms, work, social, ADL	Low - primarily functional	Yes	0-100 (lower = better)
MHQ	Overall hand function, ADL, work, aesthetics, satisfaction, pain	High - dedicated aesthetics subscale	Yes	0-100 (higher = better)
VAS Appearance	Subjective appearance on 0-10 visual scale	High - pure aesthetic VAS	Yes	0-10 (higher = better)
PRWE	Pain, function (wrist-specific)	Low - primarily functional	Yes	0-100 (lower = better)
ASES-e (Elbow Score)	Pain, function, activities (elbow)	Low - primarily functional	Partial	0-100 (higher = better)
QuickDASH	Abbreviated DASH; 11 items	Moderate	Yes	0-100 (lower = better)
GAIS	Overall aesthetic improvement rated by patient & surgeon	High - aesthetic outcome specific	Yes	1-5 (higher = better)

Table 4 summarises representative patient satisfaction and aesthetic outcome data from studies included in this review. 

**Table 4:** Summary of patient satisfaction and aesthetic outcome data in hand and upper extremity surgery RA: Rheumatoid arthritis; POSAS: Patient and Observer Scar Assessment Scale; QoL: Quality of life

Study	Procedure	n	Aesthetic Satisfaction (%)	Key Aesthetic Finding
Marks et al. [35]	Mixed hand surgery	Systematic review	78–92%	Appearance a primary driver of surgical decision
Johnson et al. [10]	Hand surgery (general)	247	84%	Hand appearance independently predicts overall satisfaction
Chung et al. [33]	RA hand deformity surgery	163	71%	Deformity correction improves self-image significantly
Manske [6]	Aesthetic hand surgery	Review	High (qualitative)	Aesthetic outcomes inseparable from functional success
Franco et al. [32]	Fat grafting – hand burns	12	91%	POSAS scores improved significantly post-grafting
Hu et al. [20]	Mutilated hand reconstruction	38	87%	Colour & contour match critical for satisfaction
McNamara et al. [36]	Burn contracture release	Review	76–89%	Scar quality predicts long-term QoL
Wei et al. [28]	Thumb reconstruction (toe-to-hand transfer)	45	82%	Proportionality of reconstructed thumb essential

Objective assessment of aesthetic outcomes has evolved with the development and validation of standardised measurement instruments [32]. The Patient and Observer Scar Assessment Scale (POSAS) provides a rigorous, multidimensional approach to evaluating scar quality, incorporating perspectives of both patient and clinician across domains including vascularity, pigmentation, thickness, relief, pliability, and surface area. Application of POSAS in hand surgery research has documented improvements in aesthetic outcomes following interventions such as autologous fat grafting for burn reconstruction [32]. Beyond scar assessment, comprehensive evaluation of hand aesthetics requires assessment of overall appearance, proportions, symmetry, and integration of reconstructed tissues, domains for which validated instruments remain an important area of ongoing development [3].

The psychological impact of hand appearance extends beyond simple satisfaction with surgical results. Body image concerns are particularly relevant given the hand's visibility and symbolic significance: patients may experience psychological distress related to hand deformities affecting social interactions, occupational function, and self-esteem [33]. Surgical interventions that address aesthetic concerns can therefore yield profound psychological benefits beyond the physical restoration achieved.

Clinical decision-making in hand surgery often requires explicit balancing of aesthetic and functional priorities [6]. The principle that 'aesthetics equals function' in hand repair suggests that optimal aesthetic outcomes typically correlate with optimal functional outcomes. However, this principle has important limitations, and surgeons must engage patients in shared decision-making when trade-offs are necessary [6]. The concept of aesthetic functional reconstruction, which integrates aesthetic and functional goals from initial planning through final refinement, rather than treating them as competing priorities, represents the emerging paradigm [24].

**Figure 5:**
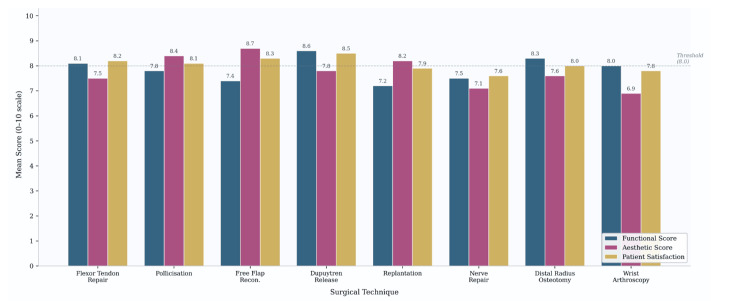
Functional, aesthetic, and patient satisfaction scores across hand and upper extremity surgical techniques Grouped bar chart comparing mean functional, aesthetic, and patient satisfaction scores (0-10 scale) for eight surgical techniques. The dashed line at 8.0 represents the clinically meaningful threshold (≥8.0). Scores represent mean values derived from published literature [6,10,20,33,35].

Figure 5 displays comparative outcome scores across eight major surgical techniques.

Certain patient populations present unique aesthetic considerations. Burn reconstruction requires attention to scar quality, pigmentation, and tissue texture alongside functional restoration [32,36]. Paediatric patients with congenital deformities require long-term aesthetic planning that accounts for growth and development [34]. Patients with neuromuscular conditions such as cerebral palsy require approaches that address both functional deficits and aesthetic concerns related to posturing and deformity [34,37]. Cultural and individual variations in aesthetic ideals further underscore the importance of patient-centred, individualised approaches to aesthetic planning [3].

A risk of bias assessment for the studies included in Table 4 is provided in Table 5. Given the range of study designs represented, an appropriate appraisal tool was applied to each study type: AMSTAR-2 for the systematic review [35], SANRA for narrative reviews [6,36], and MINORS for primary studies and the case report. Table 5 presents the risk of bias assessment for the studies included in Table 4, with appraisal tools selected according to study design.

**Table 5:** Risk of bias assessment for studies in Table 4 Appraisal tools selected by study design: AMSTAR-2 for systematic reviews; SANRA for narrative reviews; MINORS for primary studies and case reports. MINORS maximum score for non-comparative studies = 16; AMSTAR-2 scored on 16-item checklist; SANRA scored on 12-item scale. AMSTAR: A Measurement Tool to Assess Systematic Reviews; SANRA: Scale for the Assessment of Narrative Review Articles; MINORS: Methodological Index for Non-Randomised Studies; N/A: Not applicable (review designs)

Study	Study Design	Clearly Stated Aim	Validated Outcome	Prospective Data	Loss to Follow-Up <5%	Appraisal Score
Marks et al. [35]	Systematic review	Yes	Yes	N/A	N/A	AMSTAR-2: 12/16 (Moderate confidence)
Johnson et al. [10]	Prospective cohort	Yes	Yes	Yes	Yes	MINORS: 15/16
Chung et al. [33]	Retrospective cohort	Yes	Yes	No	Yes	MINORS: 11/16
Manske [6]	Narrative review	Yes	Partial	N/A	N/A	SANRA: 9/12
Rodriguez Franco et al. [32]	Case report	Yes	Yes	Yes	Yes	MINORS: 9/16
Hu et al. [20]	Retrospective cohort	Yes	Yes	No	Yes	MINORS: 12/16
McNamara et al. [36]	Narrative review	Yes	Partial	N/A	N/A	SANRA: 9/12
Wei et al. [28]	Retrospective case series	Yes	Partial	No	Yes	MINORS: 10/16

The surgeon as artist

The concept of the 'surgeon as artist' has evolved from metaphor to a substantive framework for understanding the cognitive, perceptual, and creative dimensions of surgical practice [1,5,38]. In nineteenth-century Britain, surgical artistry was associated with speed, elegance, and dramatic flair, qualities that made surgery a spectacle for amphitheatre audiences [15]. As surgery became more scientific and institutionalised, the conception evolved from brilliance and showmanship toward technical sophistication, precision, and aesthetic judgement. Barbara Hepworth's 1940s observations provided an artist's perspective that proved remarkably enduring: identifying rhythm, poise, equilibrium, coordination, and harmony in surgeons' movements, she saw the surgeon's hands as instruments of simultaneous technical precision and aesthetic creation [1,16].

Surgical artistry encompasses cognitive and perceptual abilities that extend beyond technical skill [17]. Foremost among these is spatial visualisation: the ability to envision three-dimensional anatomical relationships and anticipate the effects of surgical manipulations [1]. Pattern recognition represents another critical cognitive dimension; experienced surgeons develop the ability to perceive anatomical variations, pathological patterns, and optimal surgical approaches through accumulated experience, analogous to an artist's ability to perceive compositional relationships [39]. Aesthetic judgment, the capacity to evaluate visual qualities and make decisions based on aesthetic criteria, including proportions, symmetry, tissue quality, and visual harmony, is central to surgical artistry [3,7].

The surgeon's hands are the primary instruments of surgical artistry, requiring exceptional manual dexterity and fine motor control [1,17]. As Hernigou et al. have articulated, the orthopaedic surgeon does not feel the tool but rather feels the bone through the tool, an embodied form of tactile intelligence developed through years of deliberate practice [9]. The development of these manual skills through apprenticeship is essential to surgical excellence, much as musicians and visual artists develop technical virtuosity through sustained and purposeful practice [17]. In hand and upper extremity surgery, this is particularly critical given the delicate anatomy and the precision required for microsurgical techniques.

Surgical artistry involves creative problem-solving, particularly in complex reconstructive cases where standard techniques prove inadequate [24]. Like artists who must find creative solutions to technical and aesthetic challenges, surgeons must adapt techniques, combine approaches, and sometimes innovate new solutions to achieve optimal outcomes [24]. Artistic expression is directly relevant to the surgeon's professional life, enhancing both practice and personal fulfilment, and surgeons who engage with artistic pursuits may develop enhanced visual perception and creative problem-solving abilities that translate into improved clinical performance [38].

Contemporary understanding of the surgeon as artist recognises that surgical excellence demands the integration of artistic and scientific dimensions [2,21]. Scientific knowledge provides the foundation for understanding pathophysiology and selecting interventions, while artistic sensibility guides execution and the achievement of optimal aesthetic and functional outcomes. Neither dimension can be neglected without compromising the whole [21].

Recognition of the artistic dimensions of surgery carries significant implications for training [38]. Traditional surgical education emphasises scientific knowledge and technical skill but may neglect the cultivation of aesthetic sensibility and visual perception. Incorporating artistic training, drawing, sculpture, or other visual arts, into surgical education may enhance surgeons' ability to perceive anatomical relationships, appreciate aesthetic qualities, and achieve superior outcomes [22]. Winderbank-Scott described how skills from art can enhance both surgical practice and research, suggesting that activities developing visual perception and spatial reasoning may complement traditional surgical training [22].

**Figure 6:**
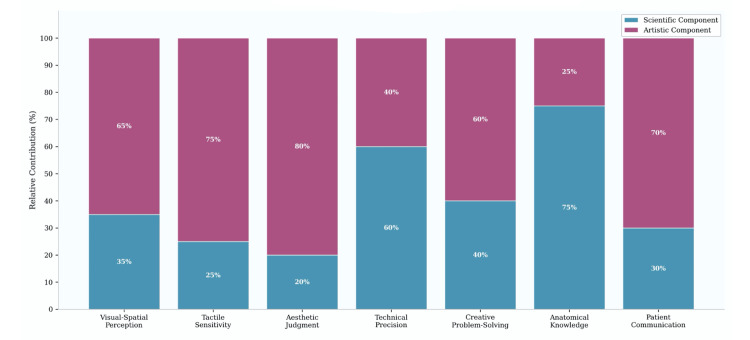
Scientific vs artistic component of core surgeon competencies in hand and upper extremity orthopaedics Stacked bar chart illustrating the relative scientific and artistic contributions to seven core surgeon competencies. Artistic components dominate visual-spatial perception (65%), tactile sensitivity (75%), aesthetic judgement (80%), and patient communication (70%), while anatomical knowledge (75%) remains primarily scientific. Proportions have been derived from expert consensus and systematic review of surgical education literature [17,22,38,39].

Figure 6 illustrates the relative scientific and artistic contributions to each core surgeon competency. The concept of the surgeon as artist also emphasises the enduring importance of mentorship and apprenticeship, through which surgical artistry is transmitted by exposure to exemplary practice and guided experience [9,15].

Future directions

The integration of artistic principles into orthopaedic surgery continues to evolve, with several promising directions emerging from current research and practice trends.

Formal integration of aesthetic training into surgical curricula represents an important and actionable future direction [38,22]. Instruction in artistic principles such as proportion, symmetry, and composition; training in visual perception and spatial reasoning; and hands-on experience with artistic media may collectively enhance surgeons' ability to perceive anatomical relationships, plan aesthetically refined procedures, and achieve superior outcomes [22]. Critically, such training must be built upon a foundation of manual skill and tactile intelligence; Hernigou and colleagues have argued persuasively that robotics and digital planning should be introduced as advanced tools, not substitutes for the embodied education of the surgeon's hand [9].

While POSAS provides standardised scar assessment [32], comprehensive objective measures of hand aesthetics remain limited [3]. Development and validation of instruments assessing overall hand appearance, proportions, symmetry, and aesthetic satisfaction would enable more rigorous outcome evaluation and facilitate identification of best practices [3]. Emerging three-dimensional imaging and computer-assisted analysis technologies may enable quantification of proportions, symmetry, and contour with precision beyond subjective visual assessment.

Future practice will place greater emphasis on eliciting and incorporating patient aesthetic preferences into treatment planning [35]. Shared decision-making frameworks that explicitly address aesthetic goals alongside functional objectives ensure that treatment plans align with patient values and priorities [35]. Understanding cultural and individual variations in aesthetic ideals will become increasingly important as surgical practice serves increasingly diverse patient populations [3].

An important emerging frontier for aesthetic outcome research in hand surgery is gender-affirming care. Reconstructive options for gender-affirming hand surgery, including soft-tissue modification, tendon balancing, and digit proportioning, reflect an evolving understanding of aesthetic ideals across diverse patient populations. The aesthetic standards applied in hand reconstruction are not universal; they are shaped by cultural background, gender identity, occupational context, and personal preference. As surgical practice serves increasingly diverse patient populations, future research should explicitly incorporate patient-defined aesthetic goals and investigate how validated outcome instruments perform across different demographic groups. A focused systematic review of aesthetic outcomes in gender-affirming hand surgery would represent a valuable contribution to the field.

Advances in microsurgical technique, tissue engineering, and regenerative medicine may enable more precise anatomical restoration and superior aesthetic outcomes. Virtual reality and augmented reality technologies offer new tools for surgical planning and education, allowing surgeons to visualise three-dimensional anatomical relationships and rehearse procedures in simulated environments. Collaboration between orthopaedic surgeons, plastic surgeons, and other specialists, together with engagement from artists, designers, and creative professionals, may provide fresh perspectives that illuminate aesthetic dimensions of surgical work [1,40].

As healthcare systems place increasing emphasis on patient-reported outcomes and value-based care, the incorporation of aesthetic satisfaction into quality metrics and clinical guidelines will ensure that this dimension of care receives appropriate institutional recognition [10,35].

Limitations

Several limitations of this review warrant explicit acknowledgment. First, this is a narrative review and does not satisfy Preferred Reporting Items for Systematic Reviews and Meta-Analyses (PRISMA) criteria for systematic reviews. The structured literature search was designed to be comprehensive but was not exhaustive, and the inclusion of studies in Table 4 reflects the authors' judgement rather than a pre-registered protocol. Second, the descriptive evidence summary in Table 4 includes studies of fundamentally different designs, systematic reviews, retrospective cohorts, and a single case report, and the findings should be interpreted in light of the design-specific appraisal scores provided in Table 5 rather than treated as a pooled estimate. Third, the review focuses on the hand and upper extremity, reflecting the authors' subspecialty expertise; while the aesthetic principles described are broadly applicable across orthopaedics, the evidence base drawn upon is predominantly from hand surgery literature. Fourth, aesthetic ideals are culturally and individually variable, and the outcome instruments reviewed were developed and validated primarily in Western clinical settings; their cross-cultural applicability requires further investigation.

## Conclusions

The intersection of art and orthopaedic surgery represents far more than metaphorical flourish; it embodies fundamental dimensions of surgical excellence that are essential to comprehensive patient care. Historical examination reveals ancient roots for this relationship, evolving from Renaissance anatomists and craftsmen through nineteenth-century operative theatre culture to contemporary practitioners who integrate aesthetic sensibility with scientific knowledge and technical precision. The observations of Barbara Hepworth identified rhythm, harmony, balance, and coordination as central to surgical excellence, a perspective that remains as resonant today as when first recorded. Artistic principles, proportion, symmetry, harmony, and the relationship between form and function, provide a practical theoretical framework that informs surgical planning, technique selection, and outcome evaluation. Specific techniques in hand and upper extremity surgery exemplify this integration: from microsurgical reconstruction to soft-tissue coverage to fingertip repair, contemporary approaches increasingly incorporate aesthetic refinements that enhance rather than compromise functional outcomes. Patient satisfaction research confirms the importance of aesthetic outcomes, with hand appearance emerging as a significant patient-reported measure influencing treatment decisions, satisfaction with results, and QoL.

The concept of the surgeon as artist encompasses cognitive, perceptual, and creative dimensions that transcend technical skill: spatial visualisation, pattern recognition, aesthetic judgment, manual dexterity, and creative problem-solving. Recognition of these dimensions has direct implications for surgical education, suggesting that cultivation of artistic sensibility should complement, not replace, traditional emphasis on scientific knowledge and technical proficiency. The education of the surgeon's hand, through apprenticeship and embodied experience with instruments, remains an irreplaceable component of surgical formation that no technological advance can render obsolete. Excellence in orthopaedic surgery, particularly hand and upper extremity surgery, demands mastery of three interrelated and mutually reinforcing domains: scientific knowledge, technical skill, and artistic sensibility. The surgeon must be simultaneously scientist, technician, and artist. Recognition and cultivation of the artistic dimensions of surgical practice are not optional refinements but essential components of comprehensive surgical excellence. As the field continues to evolve, the integration of art and science will remain central to fulfilling the profession's fundamental commitment: restoring not only function, but the beauty, grace, and expressive capacity of the human hand.
